# Developing insights from the collective voice of target users in Twitter

**DOI:** 10.1186/s40537-022-00611-5

**Published:** 2022-06-02

**Authors:** Kang-Pyo Lee, Suyong Song

**Affiliations:** 1 Department of Business Analytics, Tippie College of Business, University of Iowa, Iowa City, IA 52242 USA; 2 Department of Economics and Department of Finance, Tippie College of Business, University of Iowa, Iowa City, IA 52242 USA

**Keywords:** Twitter, User profiling, Social trends, Big Data, Text analysis

## Abstract

This study develops a pragmatic scheme that facilitates insight development from the collective voice of target users in Twitter, which has not been considered in the existing literature. While relying on a wide range of existing approaches to Twitter user profiling, this study provides a novel and generic procedure that enables researchers to identify the right users in Twitter and discover topical and social insights from their tweets. To identify a target audience of Twitter users that meets certain criteria, we first explore user profiling, potentially followed by text-based, customized user profiling leveraging hashtags as features for machine learning. We then present how to mine popular topics and influential actors from Twitter data. Two case studies on 16 thousand young women interested in fashion and 68 thousand people sharing the same interest in the Me Too movement indicate that our approach facilitates discovery of social trends among people in a particular domain.

## Introduction

Rich multidisciplinary literature has shown that Twitter data can be adapted to develop useful indicators for social trends. Few studies, however, propose a unified scheme that provides researchers with detailed and practical guidance for discovering social insights. The goal of this paper is to fill the gap. This paper suggests a comprehensive perspective to utilize Twitter data for text-based, customized user profiling, which can serve as an alternative to the existing user profiling methods, and to develop effective social trends from the collective voice of target users.

With the new opportunities brought by the emergence of Big Data into traditional survey research [1], social media have been considered a good source for public opinion research and social trend analysis. Popular social media services such as Twitter and Facebook are known for their open nature that allows people to freely share their opinions, attitudes, and behaviors.

One of the remarkable features of Big Data created from social media is that they provide “organic data”, as opposed to “designed data”, as stated by Groves [2]. Traditional surveys analyze designed data, or “made data”, which were initially made through the intervention of researchers and thus carefully designed to help answer the research question. In contrast, organic data, also known as “found data”, are not originally made to answer research questions. They were intended for another primary use and just found by researchers regardless of the original intention of the data. Social media data are a good example of this; most of the social media services we use everyday were never designed for research. Simply because they were not originally made for research, there is no guarantee that the found data can help to answer a research question.

Due to this naturally occurring nature of social media data, the question of what research areas could benefit from the organic data has been extensively addressed in many different sectors such as academia, industry, and governments in the last decade, one of which is social trend analysis. As with any type of Big Data, social media data tend to become more significant when aggregated in a large scale, and the collective voice from social media can serve as powerful indicators that signal social trends in a market or a society. What many people say on social media can be considered their interests, which can translate into a certain social trend.

A traditional survey begins by establishing study objectives, defining a target population of interest, and then selecting a sampling frame, or a survey population to interview. This sample is expected to represent the entire target population substantially, if not completely. These initial steps can equally apply to social trend analysis leveraging social media. The selection of users from social media depends on who should be targeted at in the study. For example, suppose that a market research project aims to discover new social trends among young women who are interested in fashion. To that end, a team of researchers opt to look at Twitter and collect a large amount of Twitter data to create a pool of random Twitter users and tweets. To select the right users for this study from the pool, it is essential that they need to know the age, gender, and interests of each user in the pool, so that they can identify young female users interested in fashion. This process is called user profiling, or user modeling. User profiling aims to identify a set of attributes of users that are essential to the study, such as demographic attributes (e.g., age and gender) and any other personal attributes that are helpful to know for the study (e.g., interests and personal traits). The more we know about users, the more effective user targeting will become. It is only when we can identify the right users on social media that we are able to discover social trends from the target users. In other words, detecting social trends would not make sense if we fail to identify the target users who are believed to represent the target population for the study. Previous literature has been focused on this user profiling task from many different perspectives, which will be presented in detail in the next section.

Choosing the right social media platform is another essential aspect of user targeting, as it determines the pool of candidate users. Of the many existing social network platforms that can be characterized in different ways as listed by Musial et al. [3], Twitter has been gaining the most attention from researchers primarily due to its topological characteristics in the form of follower-followee relationship and also its power as a new medium of information sharing [4]. Its open nature allows people to talk about anything and everything on Twitter, except for some unusual cases when it does harm to the public. This open nature offers researchers unprecedented opportunities to have a better understanding of people from what they share online with the world. In addition, Twitter opens part of its user-created data to the public in the form of Application Programming Interface (API), called Twitter API.^1^ For example, Twitter Streaming API, which allows users to retrieve real-time tweets from Twitter, is known to provide up to 1% sample of all the tweets created on Twitter at a given time.^2^ While this 1% sample may appear to be too small to be used in a study, it could be sufficient in many cases, considering the enormous size of the entire data. On the other hand, it is known that the random samples from Twitter could have a potential bias [5].

[6] presents a novel error framework for Twitter opinion research called Total Twitter Error, which is a variation on the traditional Total Survey Error [7] that was originally designed to conceptualize the procedural and statistical errors of survey estimates. Specifically, the Total Twitter Error framework comprises three broad error sources: coverage error (over- and under-coverage of Twitter users and tweets), query error (inaccurate search queries leading to failure to extract proper data for analysis), and interpretation error (discrepancy between the true value or meaning and the one inferred from the interpretation). These three types of errors will be mentioned wherever possible and necessary in this paper.

There has been a wide range of research that attempts to identify social trends represented on social media, and each study has its own ways to collect and process data to detect trends. Few studies, however, provide a generic procedure that guides researchers who want to leverage social media data, more specifically Twitter data, for social trend analysis. This study has two main objectives: (1) to effectively identify the target audience of users in Twitter data by user profiling and (2) to develop topical and social insights from the collective voice of the target users. For the user profiling task, specifically, we present text-based customized user profiling, which can be considered to be an alternative when there are no existing user profiling solutions that are available or work for the user attribute or the data of interest. We believe that this study is novel in that it presents a pragmatic scheme for Twitter user profiling and social trend discovery with a comprehensive and detailed guidance on how to use raw Twitter data to identify the target audience for a study and mine social trends from what the target users say on Twitter.

Two case studies support that our approach facilitates discovery of social trends among a group of people on Twitter in a particular domain. The first case study identifies a target audience of young female users who are interested in fashion and successfully discovers the popular topics and influential actors among them, which are believed to provide insights into marketing strategies. For user profiling, we apply heuristics for the interest attribute of users as well as some of the available user profiling solutions that proved to perform well for the account type, gender, and age attributes. The second case study demonstrates that political orientation, i.e., conservative vs. liberal, does affect the reactions to the Me Too movement. Leveraging customized user profiling to identify the political orientation of each user, we develop our own high-performing political orientation classifier from the Random Forest﻿ algorithm, which is fitted to our Twitter data.

There have been recent research papers whose application of sentiment analysis has been extended to many practical fields from medicine to economics. For instance, [8] show how data posted on Facebook by Crohn’s disease patients are can be used to understand the patient’s perspective on a given medical prescription. [9] show that an economic sentiment derived from economic and financial newspaper articles is predictive of movements of survey-based measures of consumer sentiment. Similarly, [10] use a self-attention-based model to measure business sentiment based on textual data from daily newspaper articles. They show that the proposed index is strongly correlated with established survey-based index and a variety of economic indices. Even though the current study primarily focuses on Twitter data, the proposed text-based approach has a potential that can be extended to other text data analysis in order to develop sentiment indexes for many disciplines.

The rest of this paper is organized as follows. "Related literature" section outlines related literature on user profiling. "Discovering social trends in a target audience" section describes the steps for Twitter user profiling and social trend discovery. "Case studies" section discusses two in-depth case studies: one on women’s fashion market research and the other on the Me Too movement reaction. "Discussion and conclusions" section concludes and offers some directions for future research.

## Related literature

User profiling has been known as an effective way to gain a better understanding of users in a platform, and the enhanced understanding of users can facilitate many different applications such as target marketing and personalized recommendation. It is worth noting that the majority of studies on user profiling chooses Twitter among many other social media platforms, primarily due to its open and data-friendly nature, which was previously discussed in "Introduction" section.

User profiling focuses on what attributes of users need to be identified. User attributes can be categorized into two broad categories: demographic attributes and other personal attributes. Demographic attributes of users have been extensively addressed as the primary information about users, due to the fact that they tell much about someone. Demographic attributes include age, education, gender, location, marital status or spouse, language, and race or ethnicity. There are other personal attributes including account type (personal vs. organizational or human vs. bot), expertise, hobbies, interests, personal traits, political orientation, and influence. Table 1 lists the user attributes that can be inferred from Twitter data and the proposed methodologies for each user attribute. Note that the list of methodologies in the table is not exhaustive due to the vast amount of literature.

**Table 1:** Summary of the derivable user attributes, necessary data, and existing methodologies

Type	User attribute	Data (Methodology)
Demographic attributes	Age	Tweet text ([11]) Tweet text, follow ([12]) Profile image, the **name**, **screen name**, and **description** fields in **User** object ([13])
	Education	Tweet text, follow ([14])
	Gender	The **name** field in **User** object ([18] Profile image, the **name**, **screen name**, and **description** fields in **User** object ([13]) Tweet text ([11, 15–17]) Tweet text, follow ([12])
	Location	The **location** field in **User** object ([18] Tweet text ([11, 21–26]) Tweet text, follow ([12, 27]) Tweet text, tweet context ([28, 29])
	Marital status /spouse	Tweet text, follow ([12, 14, 30])
	Language variety	Tweet text ([15, 17])
	Occupation	Tweet text ([31]) Tweet text, follow ([12, 14])
	Race/ethnicity	The **name** field in **User** object ([18] Tweet text, **User** object field, follow ([32])
Other personal attributes	Account type	Tweet text ([16]) Tweet text, follow ([30]) Tweet text, **User** object fields ([33]) **User** object fields, tweet context ([34]) Profile image, the **name**, **screen name**, and **description** fields in **User** object ([13])
	Expertise	Tweet text, the **description** field in **User** object, user lists ([35]) User lists ([36])
	Hobbies	Tweet text, follow ([12])
	Interests	Tweet text ([37–39]) Tweet text, follow ([40, 41]) Posted URLs ([42]) User lists ([36])
	Personality traits - Big Five	Tweet text ([43–47]) **User** object fields ([48])
	Personality traits - Dark Triad	Tweet text and **User** object fields ([49])
	Personality traits - MBTI	Tweet text ([50, 51])
	Political orientation	Tweet text ([11, 52]) Tweet text, **User** object fields, follow ([32])
	Influence	Follow ([55–57]) Follow, tweet text ([58, 59]) Tweet text ([60])

Regarding the age attribute, since it is challenging to identify the exact age of a user, previous work has been focused on identifying predefined age ranges, e.g., below 30 vs. above 30 [11] or 10s or younger vs. 20s vs. 30s vs. over 40s [12, 13]. Rao et al. [11] consider only the tweet text of users for age identification, whereas [12] consider both follow relationship of users and tweet text. More recently, [13] utilize the profile image and the **name**, **screen name**, and **description** fields in a **User** object to identify the age as well as the gender and account type with a single multi-modal model. This technique will be used in our first case study in "Case studies" section.

Identifying education level and spouse of users has not been extensively addressed mainly due to the lack of available training data, as stated by [14]. The study employs a technique called distant supervision which learns to extract relations from text using ground truth from an existing database such as Freebase, to detect school and spouse entities mentioned in tweet text.

For gender classification, most of the studies such as [11, 15–17] consider tweet text, based on the idea that user’s gender with only two classes, female and male, can be distinguished from what they say and the way they say on Twitter. Mislove et al. [18] simply consider the **description** field of a **User** object, while [12] consider follow relationship as well as tweet text.^3^

User location is one of the attributes that have been investigated the most extensively for many different purposes. Here, locations refer to users’ home locations indicating their residences, tweet locations as their current locations at the time of tweet posting, and mentioned locations reflecting their places of interest. Zheng et al. [19] provide a comprehensive survey of the existing approaches to location prediction on Twitter. Most of the studies are motivated by the fact that only a small portion of tweets are geo-tagged or geo-referenced [20], which means that few tweets contain exact geo-information to be used for accurate location identification. Refs. [21, 11, 22–26] only consider tweet text for location prediction, whereas [27] and [12] add follow relationship and [28 ,29] add the tweet context as additional features of their models. [18] simply use the **location** field of a **User** object.

Marital status, i.e., whether a user is single or married, is another demographic attribute that tells much about an individual and their family. Both [12, 30] consider tweet text and follow relationship for marital status identification.

For language variety identification, which can also be related to race or ethnicity of a user, [15] identify for four languages, English, Spanish, Arabic, and Portuguese, while [17] distinguish two languages, English and Spanish, both considering tweet text.

Identification of occupation is motivated by the fact that a person’s life is deeply connected with and explained by their occupation. Hu et al. [31] consider eight job categories such as Marketing, Administrator, Start-up, Editor, Software Engineer, Public Relation, Office Clerk, and Designer. Ikeda et al. [12] consider seven job categories including Employee, Part-time, Self Employed, Civil Servant, Homemaker, Student, and Without occupation, while [14] identify specific job entities in tweets. Hu et al. [31] use tweet text, whereas [12, 14] use follow relationship as well as tweet text.

Race or ethnicity has not been addressed as much as other demographic attributes. Mislove et al. [18] consider the **name** field of a **User** object to extract the last names of users and compare them with the U.S. 2000 Census data. Pennacchiotti et al. [32] consider tweet text, some fields in **User** object, and follow relationship of users to identify whether a user is either African-American or not.

In addition to demographic attributes, there are other personal attributes that can be identified by user profiling. Account type identification is interesting in that it aims to tell whether a user account on Twitter is either a personal account or not, in other words, an organizational account or a bot account. Fagni et al. [16] consider tweet text to first identify whether an account is either human or bot, and, in case of human, further identify the gender (female vs. male). Oentaryo et al. [30] use tweets and follow relationship to identify whether an account is either personal or organizational. McCorriston et al. [33] address the same problem using some fields in **User** object. Alzahrani et al. [34] focus on detecting only organizational accounts using some fields in **User** object and tweet context.

Expertise is another interesting attribute in that it can be used for applications such as personalized recommendation, expertise matching, and community detection. Refs. [35, 36] both use user lists which are curated groups of Twitter accounts created and managed by users, while the former additionally use tweet text and the **description** field of a **User** object to extract expertise.

Regarding hobbies [12], is the only study we have found, which identifies the hobbies of Twitter users from the twelve hobby categories such as Reading, Gourmet, Vehicle, IT & Electronics, Games, Pets & Plants, Sports, Travel, Fashion, Music, TV & Movie, and Arts, by considering tweet text and follow relationship.

Interests are among the most extensively investigated user attributes along with the location attribute, as users’ interests can be directly used for applications such as personalization and customized marketing. The literature ranges from the studies considering only tweet text [37–39]) to those considering follow relationship as well as tweet text [40, 41], the one considering only the posted URL in tweets [42], and the one considering user lists [36].

Identification of Personality traits attempts to classify users’ personality into one of the well-known personality trait categories such as Big Five (Openness, Conscientiousness, Extroversion, Ageeableness, and Neuroticism), Dark Triad (Narcissism, Machiavellianism, and Psychopathy), Myers-Briggs Type Indicators, or MBTI. The Big Five model has been adopted by most of the studies such as [43–48], while there are a study focusing on the anti-social traits called Dark Triad [49] and studies adopting MBTI [50, 51].

Identification of political orientation, affiliation, or preference has been addressed as a binary classification problem with only two classes: Republican/conservative/right vs. Democratic/liberal/left. Refs. [11, 52] consider only tweet text, whereas [32] consider some fields in **User** object, follow relationship, and tweet text.

Last, user influence refers to the influence of a user on other users in a social network. This measure can be leveraged to identify influencers or opinion leaders in a domain. Here, measuring how influential someone is can be very subjective, which has lead researchers in many different disciplines such as social science and economics to propose a variety of approaches to measuring user influence. Refs. [53, 54] provide great overviews of the existing influence measures for Twitter users in literature. Refs. [55–57] only rely on the follow relationship to apply traditional centrality measures such as closeness, betweenness, and PageRank to Twitter users, whereas [58, 59] add tweet text as an additional source to consider and [60] utilize tweet text alone to measure user influence.

## Discovering social trends in a target audience

### Methodology

We present the details of how to discover a target audience of Twitter users and their collective voice from raw Twitter data. First, in order to identify candidate users that meet certain criteria, we explore available Twitter resources for data collection and existing approaches to user profiling. Next, we discuss enriching user profiles utilizing hashtags in the tweets posted by the target users. Lastly, we present developing topical and social insights from the collective voice of the target users.

Before we go into details, we first present formal modeling of the data space that we analyze in this paper. Our Twitter data space can be noted as ${\mathcal {U}} \times {\mathcal {T}} \times {\mathcal {H}}$, where ${\mathcal {U}}$ is a set of users on Twitter, ${\mathcal {T}}$ is a set of tweets created by the users, and ${\mathcal {H}}$ is a set of hashtags used in the tweets by the users. This implies that a user $u \in {\mathcal {U}}$ creates a tweet $t \in {\mathcal {T}}$ using a set of hashtags ${\mathcal {H}}_{u,t} \subset {\mathcal {H}}$.

User profiling is an essential component to our approach, which defines user attributes needed for a study and populates the attribute values for each user. We define the profile of a Twitter user $u \in U$ as a set of tuples consisting of an attribute and its value where, with respect to user *u* for an attribute $a \in A$, its value *p*(*u*, *a*) is computed by a user profiling function *p*, as in Eq. (1):

1 $$\begin{aligned} P_{u}=\{(a,p(u,a)) \mid a\in A, u\in U\}, \end{aligned}$$

where *A* is a set of user attributes. Determining the user profiling function *p* for each user attribute is the goal of the user profiling phase.

**Fig. 1:**
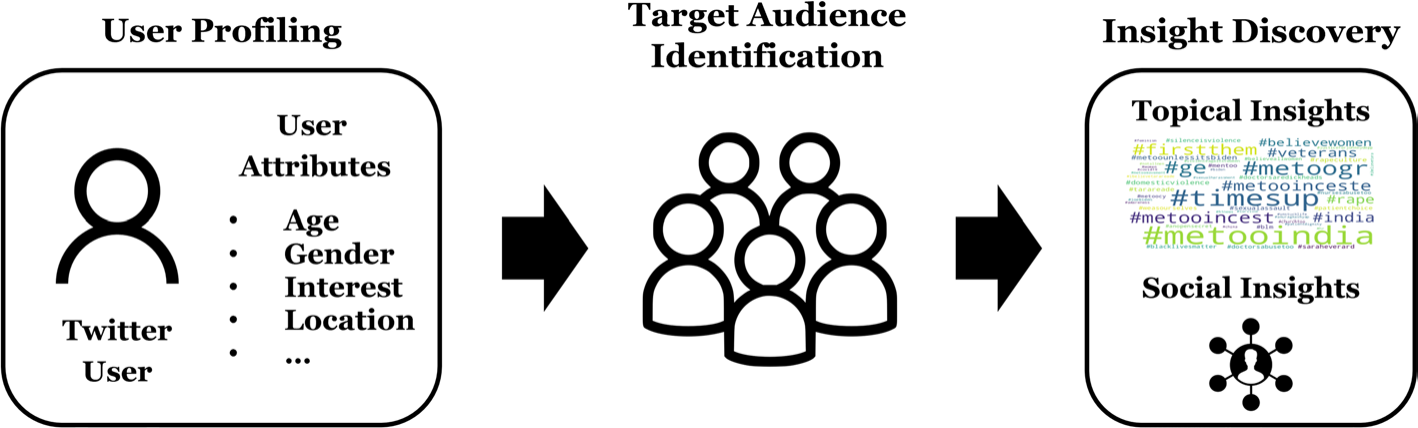
The flow map of our unified scheme for developing social insights from the collective voice of target users

Figure 1 illustrates the flow of our unified scheme for developing social insights from the collective voice of target users. First, attributes of Twitter users are identified in the user profiling stage such as demographic attributes and other personal attributes. When some user attributes are missing due to data availability, researchers can consider developing their own customized solution to a specific user profiling task. A supervised machine learning model can be built by utilizing hashtags as the features for prediction. Second, once this user profiling phase is completed, researchers select only the users of interest based on the identified user attributes. Finally, researchers proceed to develop topical and social insights from the collective voice of these target users.

### User profiling

In general, sampling of Twitter users is less common than sampling of tweets due to the limited functionality of Twitter API for collecting users. For this reason, we begin with a large pool of random tweets, which are known to be much easier to collect via Twitter API mentioned earlier in "Introduction" section. Each tweet collected contains author information describing the user who created the tweet. Some user attributes for the users in the pool are already known or can be easily acquired, while other attributes need to be inferred, are difficult, or impossible to identify. It is worth noting that raw user data collected from Twitter via Twitter API provides surprisingly useful information about users. Table 2 lists native Twitter objects and their fields along with user attributes that can be derived from the fields. Twitter API provides several types of objects encoded in JavaScript Object Notation (JSON), of which **User** and **Tweet** objects are the most useful in user profiling.^4^

**Table 2:** Summary of the user attributes derivable from native Twitter objects

Object	Field	Description	Derivable user attributes
User	name	Name of the user	Name, gender, age, race/ethnicity
	location	User-defined location for the account’s profile	Location
	url	URL provided by the user in association with their profile	Web site, blog, or other social media accounts
	description	User-defined description of their account	Demographics, expertise, hobbies, interests, personality traits, political orientation
	verified	Whether Twitter has verified that the account of public interest is authentic	Popularity
	followers_count	Number of users following the account	Popularity
	friends_count	Number of users the account is following	Sociability
	listed_count	Number of public lists that the user is a member of	Popularity
	favourites_count	Number of tweets the user has liked in the account’s lifetime	Posting activeness
	statuses_count	Number of tweets (including retweets) issued by the user	Posting activeness
	created_at	UTC datetime that the user account was created on Twitter	Account age
	profile_image_url_https	HTTPS-based URL pointing to the user’s profile image	Gender, age, race/ethnicity
	followers*	List of users following the account	Network
	friends*	List of users the account is following	Network
Tweet	created_at	UTC time when the tweet was created	Behavior
	text	Actual text of the status update	Demographics, expertise, interests, personality traits, political orientation
	coordinates	Geographic location of the tweet as longitude and latitude coordinates	Location, behavior
	place	Known place as city, state, or country	Location, behavior
	reply_count	Number of times the tweet has been replied to	Popularity
	retweet_count	Number of times the tweet has been retweeted by other users	Popularity
	favorite_count	Number of times the tweet has been liked by other users	Popularity
	lang	Machine-detected language of the tweet	Language
	retweeted_status	Original tweet object if the tweet is a retweet	Typical tweet or retweet

A **User** object, which describes an individual user on Twitter, has several fields that can be directly used as user attributes, such as name, location, and url, while the other fields can be analyzed to infer new attributes.^5^ For example, from the **description** field that has a user-defined description or bio of an account, one can infer many different types of user attributes, such as demographic attributes (e.g., age, education, gender, location, marital status, language, occupation, and race/ethnicity) and other personal attributes (e.g., expertise, hobbies, interests, personality traits, and political orientation), depending on the information included in the text of the field. A wide range of natural language processing (NLP) and text mining techniques can be applied to this field. The other fields in a **User** object can be good indicators of the account’s popularity, sociability, or activeness. For example, the **followers_count** and the **listed_count** fields indicate how popular the account is, while the **friends_count** field indicates how sociable the account is. One may want to compare the **followers_count** to the **friends_count**, to see if there is a large or small gap between the two fields. For example, celebrities tend to have a very large number of followers but a smaller number of friends, whereas spam accounts or bot accounts tend to have many friends but few followers.

The **favourites_count** and the **statuses_count** fields can be used to measure how active the account is in terms of posting tweets. The **created_at** field can be used to calculate the account age in days, months, or years, which can be combined with other fields for normalization. For example, users who have been using Twitter for ten years would probably have more followers or have posted more tweets than those who just began to use Twitter. In this case, one may need to divide the number of followers or number of statuses by the account age, so that the indicators can be normalized for each user.

A profile image from the **profile_image_url_https** field can be used to identify gender, age, or race/ethnicity of the user by applying state-of-the-art image analysis techniques [13, 61]. The **followers** field contains the lists of users following the account, while the **friends** field contains the list of users the account is following, both of which present the relationship network of the user. Note that the two fields, each marked with an asterisk, are not actually linked to the **User** object as its fields. Twitter API separates these two fields from the **User** object for some reason. But we link them as fields of the **User** object, as we believe those fields should also be treated as user attributes.^6^ The two fields provide direct information about who are the followers and friends of a user. The **verified** field is a unique feature of Twitter, which indicates whether Twitter has verified that the account of public interest is authentic.^7^ A verified account has a blue verified badge on Twitter. This can serve as another indicator of the user’s popularity or authority.

A **Tweet** object describes an individual tweet posted by a user.^8^ An individual tweet could not be directly used as an attribute of a user due to its limited information. When aggregated, however, they can be a powerful source for a researcher to understand the user. While a **Tweet** object has a number of fields, the bottom half of Table 2 lists a few of those that can be used to infer user attributes. The **text** field is the most important one among all fields, as it provides raw tweet text written by the user. It is worth noting that tweet text can have up to only 280 characters (the length limit was increased from 140 to 280 in 2017), which is why Twitter is called a micro-blogging service. The short text has its own pros and cons. In some cases, tweet text might be too short to convey meaningful information from an analysis perspective, while in other cases a single short tweet can have enough information to understand the user. On the other hand, the short text is what has made people freely use Twitter. From a Big Data perspective, the more tweet text we have for a user, the better understanding of the user we will have. The **text** field can be used to infer most of the demographic attributes and personal attributes mentioned earlier. As with the **description** field of a **User** object, this field can benefit from text analysis techniques.

The **created_at**, **coordinates**, and **place** fields can bring a temporal or a geo-spatial aspect to the study. While every tweet has a value in its **created_at** field, not all tweets have values in the **coordinates** and **place** fields. It depends on whether the user had activated location sharing in their applications. It is known that, as already discussed earlier, only a small fraction of tweets are geo-tagged or geo-referenced [20]. The three fields **reply_count**, **retweet_count**, **favorite_count** are considered to be good indicators for the popularity of the tweet, which can also translate into the popularity of the user. The **lang** field indicates which language the user is primarily using or able to use. It is also worth noting that users can retweet other users’ tweets, and those retweets are considered to be the user’s tweets, although they were originally created by others (users can also add their own comments to the original tweet when retweeting). If we analyze tweets to understand the user, however, those retweets could be of no help, because they were not originally created by the user. In this case, by referring to the **retweeted_status** field, those retweets can be excluded from any analysis, so that only the normal tweets created by the user are considered.

The Twitter objects and their associated fields listed in Table 2 provide insight into some heuristics for user profiling before attempting to apply advanced methodologies. In particular, the **description** field of a **User** object can be directly used to extract various user attributes like gender, location, occupation, and so on. The following description from a Twitter user account, which is open to the public, is a good example:

Senior Narrative Designer @UbiMassive — cats, books, games and scones — Brit in Sweden — opinions all mine — She/her.

This short bio tells much about the user, such as gender, occupation, hobby, nationality, and location. The user is female from the phrase “She/her”; she is a narrative designer at a game company; she likes cats, books, games, and scones; she is British; she lives in Sweden. While not all Twitter users describe themselves in such detail, it is apparent that the **description** field can serve as a primary source for understanding users. In order to extract the right information from the description text, a string pattern matching technique called regular expression can be employed.

If the approaches relying on some raw user attributes provided by Twitter are too simple to work for a research study, one should consider employing advanced techniques for user profiling listed in Table 1. As described in "Related literature" section, previous works have explored different ways of profiling Twitter users. When applying the advanced methodologies, note again that different methodologies use different data for user profiling, depending on their proposed approaches. For example, to identify the location of a user, some methodologies such as [11, 21–23] consider only tweet text, whereas other methodologies such as [12, 27–29] use not only tweet text but also use follow relationship of users or tweet context. Note also that the methodologies targeted at the same user attribute do not always yield exactly the same outcome, as each methodology has its own research questions to address. Depending on objectives of the study, a subset of the user attributes listed in Table 2 can be considered in user profiling. For the market research project example mentioned in "Introduction" section, the researchers should only focus on such attributes of users as age, gender, and interest, and thus examine which methodologies would fit the data they currently have. Again, they should be aware that different methodologies use different data. Once this user profiling task is performed over all users in the data pool, they now can select only the users that meet the criteria they have set for the study. This initial set of selected users can be further analyzed to be selected as the final set of target users.

### Customized user profiling

If the user profiling task was perfectly done and ended up properly populating all user attributes needed, we can move on to selection of target users based on the user attributes. In many cases, however, it is possible that there are no resources available for some user attributes, leaving their values missing. This can happen when (1) there are no available resources at all, (2) the existing resources do not fit the data we have, or (3) the performance of the available resource is not satisfactory.

To resolve this issue, we propose to consider developing a customized solution to a specific user profiling task, especially if it is a supervised machine learning problem. For example, suppose we want to classify each Twitter user by their political orientation, i.e., conservative or liberal. While there are some available resources for political orientation classification, as listed in Table 1, one might find that those existing resources do not work well with the recent Twitter data. This leads us to consider developing our own political orientation classifier as long as we can make labeled data that can be used for training and testing machine learning models. Inspired by the observation that some Twitter users explicitly share their political orientation in their bio, we can collect a set of those users and label them as conservative or liberal. We then can use the labeled data as training data and test data for machine learning by selecting a set of features for prediction. Specifically, we propose to utilize hashtags as the features for political orientation prediction, based on the idea that conservatives and liberals are believed to be interested in different topics to some extent, thereby using somewhat different hashtags. Once a machine learning model is built, one can apply the model to populate the values in the target user attribute. While we cannot say that this approach would work for all user profiling tasks, we believe that it can work for supervised machine learning tasks, such as classification and regression, and that it can be a good complement to the existing user profiling solutions. We call this phase text-based customized user profiling, as opposed to the primary user profiling performed in the first phase, as this customized user profiling task can complement what is missing from the primary user profiling task.

In order to utilize hashtags as features for prediction, we first need to collect the tweets posted by users and mine hashtags from the tweets. The Twitter API allows researchers to retrieve up to 3200 most recent tweets of a user account, as long as the account is set to public.^9^ Alternatively, one can consider web scraping to retrieve more than 3200 tweets from an account, although this option does not provide easy access to the web data in a structured manner unlike using an API. While all words in tweets are meaningful in one way or another, we particularly focus on hashtags in tweets. A hashtag is a word starting with a hash (#) symbol as its prefix such as #metoo, #nowplaying, and #earthday. Hashtags were originally introduced by Twitter and have been used to index keywords or topics on social media, which allow users to easily follow topics of interest. As mentioned by [62], the goal of a hashtag is to facilitate search and aggregation of messages related to the same topic. With the wide adoption of hashtags on Twitter, a number of studies have investigated hashtags on Twitter. Tsur et al. [63] attempt to predict the spread of thoughts and ideas, called memes, using hashtags. Ferragina et al. [64] address hashtag relatedness and classification. Refs. [65–69] address hashtag recommendation from a personalization perspective, while [70–74] address hashtag clustering.

One of the reasons why we focus on hashtags, instead of all words or phrases in tweets, is that they are easy to handle. As users explicitly create a hashtag with the hash symbol and a hashtag allows no space in it, they are easy to extract and aggregate from text. In fact, Twitter API provides a list of hashtags identified in a tweet as a Hashtag object, thus API users do not have to extract hashtags themselves, which otherwise should be done with the help of a text analysis technique like regular expression. The main drawback to using hashtags is its sparsity; as pointed out by Godin et al. [66], not all tweets have hashtags and not all users use hashtags. Nevertheless, this sparsity can be overcome when a large number of hashtags are aggregated, mainly because of the fact that a hashtag tends to be adopted by a significant number of users who want to join a virtual community that is interested in a certain topic [75].

Once all hashtags are extracted from tweets, they are aggregated such that the total frequency for each hashtag is calculated. Based on the hashtag frequency, one can have a hashtag popularity ranking sorted by frequency in descending order. This hashtag ranking can be a basis for researchers to manually select top-*k* popular hashtags that will be used as features for prediction, where *k* can be determined empirically. When top-*k* hashtags are selected as features, their frequencies are the values that should be put into the machine learning model. This way, one can build a model that is able to predict the value of a user attribute for a user. Building a machine learning model should always be followed by evaluating the model performance, using commonly used machine learning metrics.

### Discovering social trends

Once the user profiling is completed and all values of the user attributes needed for the study are properly populated, one can now select the target users of interest, using the user attributes. For the market research example mentioned earlier, the researchers can simply select the users in their pool, who are young, female, and interested in fashion. Given that the target users have been identified, researchers can now proceed with in-depth analysis on the collective voice of these targeted users. While this final phase should completely depend on the objectives of the study, i.e., what the researchers want to know about their target audience, we focus on hashtags from a topical perspective to discover popular or rising topics among people and also on relationship networks from a social perspective to identify influencers.

Popular hashtags among the target users can be captured in a similar way that we used earlier to identify popular hashtags for the customized user profiling. A simple frequency ranking from tweets will work for popular hashtags, while one may want to consider advanced techniques to detect a trend over time with hashtags [76–78]. Influencers in a social network can be identified as well, based on the network structure among the target users. A variety of centrality measures, such as degree centrality, closeness centrality, betweenness centrality, and eigenvector centrality, can be applied, as previously mentioned in "Related literature" section.

## Case studies

Having established a procedure to identify a target audience in Twitter and discover social trends from their collective voice, we now move on to two in-depth case studies that demonstrate how a research study can benefit from our approach. The first case study will provide details on the market research project example we have mentioned throughout this paper, while the second case study performs a comparative analysis on the effects of political orientation on a gender issue.

**Table 3:** Monthly data statistics of the pools of random Twitter users and tweets

Month	User count	Tweet count
12/2021	22,569,110	133,387,546
11/2021	21,876,935	129,462,997
10/2021	22,175,272	133,334,050
09/2021	21,446,941	127,009,377
08/2021	21,708,191	133,447,209
07/2021	21,979,242	133,358,039
06/2021	21,611,226	128,414,906
05/2021	22,651,068	133,741,215
04/2021	22,138,958	129,235,713
03/2021	22,441,309	133,544,952
02/2021	21,529,017	120,703,913
01/2021	22,317,570	133,754,300
12/2020	21,107,115	120,627,976
11/2020	21,950,691	129,635,445
10/2020	21,889,317	133,221,211
09/2020	22,344,474	128,867,950
08/2020	22,643,060	133,302,754
07/2020	22,930,209	133,609,303
06/2020	22,419,694	128,885,150
05/2020	23,554,291	133,389,857
04/2020	23,420,878	129,330,138
03/2020	23,400,803	133,474,640
02/2020	21,260,800	125,029,995
01/2020	22,275,681	133,666,464
Total (Unique)	138,845,242	3,132,435,100

To identify two sets of target users for the case studies, we first need a large pool of random Twitter users and tweets, from which target users are extracted by user profiling. To that end, we rely on the Twitter Streaming API, mentioned in "Introduction" section, for large-scale data collection. The API allows users to filter real time tweets on a set of keywords. Due to its real time nature, users begin collecting tweets at the moment of calling the API in a way that relevant tweets are streaming into the computer that has called the API. While setting a set of specific keywords of interest is a normal use of the API, setting a set of extremely general words such as stop words (e.g., ‘a’, ‘an’, and ‘the’) as keywords is a commonly used trick to collect random tweets. Each **Tweet** object from the API has a **User** object, which describes the user who created the tweet, as described in "User profiling" section. This means that we can collect a set of random users from a set of random tweets. As shown in Table 3, we have collected real time tweets for two years from January 2020 to December 2021, which leads to a large-scale data collection of approximately 3.1 billion (3,132,435,100) unique English tweets posted by approximately 138.8 million (138,845,242) unique users. Note that, while these pools of users and tweets are indeed big enough to be called Big Data, they do not always have to be this big. Smaller sets of random users and tweets could be enough depending on the objectives of the study, although smaller data sets could suffer from the under-coverage error mentioned in "Introduction" section.

### Young women’s fashion market research

Marketers want to know what their customers are currently interested in and who are the influencers among them, so that they can have insights into new business opportunities and focus their marketing effort and resources on specific people who could influence others. In this in-depth case study, we aim to first identify young, female users in Twitter who are interested in fashion and then discover popular topics and influential users among them.

In order to find the target audience of female users interested in fashion, we first begin by searching our random pool of tweets for tweets that have the hashtags #fashion and #style. As mentioned earlier, each **Tweet** object has a **User** object that indicates the user who created the tweet, which allows us to identify all users in our pool who have ever used the two hashtags. Here, mentioning the hashtags is assumed to be their interest in the topic. This step can be understood as a simplified implementation of the interests attribute in Table 1. Note that, one can consider adding more hashtags as search terms that are similar to #fashion and #style such as #beauty and #clothing. The search allows us to find 111,913 users in total. Using the Twitter API, we further check if each of these users still has a valid, public account, which leaves 89,437 users.^10^ Next, we remove users whose total number of tweets posted is fewer than 100, based on the idea that we would need at least 100 tweets to understand a user by their tweets. This results in 51,276 users in total, i.e., $|U| = 51276$. We then collect up to 3200 most recent tweets from each user using the Twitter API, which totals 107,002,581 tweets, i.e., $|T| = 107002581$.

After finding users interested in fashion and collecting their recent tweets, the next step is to identify each user’s gender and age, which will allow us to select young female users. Before applying a gender classification solution, we first remove organization accounts, based on the belief that organizations do not represent our target customers. Note that researchers may want to include organization accounts if they believe organizations are worth being considered in their study. In this case study, we are only interested in individuals, especially young female users. This step can be considered as an implementation of the account type attribute in Table 1. In order to identify organization accounts, we leverage two open source solutions: one is called Humanizr provided by [33] and the other called M3-Inference provided by [13].^11^,^12^ The Humanizr looks into tweets of a user along with user information in the tweets to determine whether the account in question belongs to an individual person or represents an organization, while M3-Inference uses the profile image, name, screen name, and the bio of a user, as already stated in "Related literature" section. In case the two solutions return different outcomes for the same account, in other words, one solution classifies as an organization account, whereas the other does as an individual account, we consider a user to be an organization account when at least one of the two says it is an organization. Otherwise, the account is considered an individual account. In our data, approximately 22% (11,195 out of 51,276) of the accounts turn out to be organization accounts, which is higher than 9.4% reported by [33]. We remove those organization accounts, which leaves 40,081 users who are believed to be individual accounts.

For gender identification, we utilize a Python library called gender-guesser, which employs a statistical approach to gender classification by considering the first name of a person, as well as the M3-Inference solution already used for the account type.^13^ The gender-guesser solution returns one of the six classes: “unknown”, “androgynous”, “male”, “female”, “mostly_male”, or “mostly_female”. Here, we merge “mostly_male” into “male” and “mostly_female” into “female”, for simplicity. As mentioned in "User profiling" section, a **User** object has the **name** field that allows users to specify their name. As not all users provide their exact full name, it is possible that there is no first name in the field. Furthermore, even if there is the first name specified by the user, there is no guarantee that the first name is recognized by the solution, which is especially true for non-English names. The M3-Inference solution returns either “female” or “male” for a user. In order to merge the outcomes from the two solutions, we (1) label the users as “conflict“ when one solution returns “female” and the other “male” and (2) label the user as the one predicted by the second solution when the first solution returns “unknown” or “androgynous” and the second solution returns “male” or “female”. This results in 24,886 females, 13,910 males, and 1285 conflicts. We disregard the conflicts in our data.

For the age attribute, we continue to rely on the M3-Inference solution, which returns for each user one of the four age levels: ≤18, (18, 30), [30, 40), [40, 99). From our data, the solution results in 6,011 users for 18 or under, 12,994 for 19 to 29, 10,641 for 30 to 39, and 10,435 for 40 or above.

Now that we know all four user attributes needed for this study, i.e., interest, account type, gender, and age, we can select, from the users interested in fashion, those who are young and female. For the age attribute specifically, we define young women as those in the following two age classes: (18, 30) and [30, 40). This entire selection process of target users results in 16,011 users, who form the final target audience for this study, and 31,506,037 tweets posted by the users, which will be further analyzed. As a reminder, we identify these 16,011 young females out of all 51,276 users.

**Table 4:** Top-50 popular hashtags from the tweets posted by the young female users interested in fashion

Rank	Hashtag	Frequency	Rank	Hashtag	Frequency
1	#poshmark	4,993,200	26	#fitness	19,223
2	#shopmycloset	3,748,873	27	#nature	19,137
3	#fashion	2,351,297	28	#model	18,601
4	#style	1,569,501	29	#nyc	18,409
5	#giveaway	79,435	30	#summer	18,025
6	#love	73,497	31	#quote	17,928
7	#etsy	71,360	32	#tbt	17,591
8	#win	67,961	33	#blog	17,575
9	#shehnaazgill	57,871	34	#shopping	17,510
10	#beauty	54,519	35	#sidharthshukla	17,205
11	#handmade	48,495	36	#design	16,366
12	#art	39,531	37	#life	16,261
13	#vintage	36,432	38	#gifts	16,178
14	#jewelry	31,311	39	#sale	16,084
15	#ad	31,195	40	#covid19	16,066
16	#ootd	29,427	41	#sweepstakes	16,019
17	#beautiful	28,299	42	#android	15,903
18	#photography	27,432	43	#food	15,695
19	#travel	25,743	44	#mayward	15,661
20	#christmas	24,971	45	#androidgames	15,294
21	#makeup	24,902	46	#cute	15,289
22	#music	22,309	47	#health	15,187
23	#ebay	21,732	48	#sexy	14,926
24	#gameinsight	21,027	49	#tiktok	14,921
25	#repost	20,442	50	#contest	14,897

We now proceed with the last step for gaining insights into popular topics and influential users among the young women interested in fashion. To discover popular topics, we look at popular hashtags used by them in their tweets. When extracting hashtags from tweets, we exclude those hashtags that are exclusively used by a single user. Specifically, a hashtag is excluded if its frequency rate from the most contributing user is higher than or equal to 0.5. We also exclude non-English hashtags. Table 4 presents the top-50 popular hashtag ranking. All the hashtags on this ranking provide us with direct or indirect insights into young female users’ interests in the fashion domain. For example, the first-, second-, and seventh-ranked hashtags #poshmark, #shopmycloset, and #etsy clearly show how popular shopping on Poshmark and Etsy is among young women. Other hashtags on the ranking are also intriguing, such as #handmade, #vintage, #jewelry, #ootd (meaning outfit of the day), #makeup, and #fitness, to name a few. Marketers can get some ideas from these popular hashtags for their marketing strategies.

**Table 5:** Top-50 popular user mentions from the tweets posted by the young female users interested in fashion

Rank	User	Frequency	Rank	User	Frequency
1	@poshmarkapp	4,917,306	26	@jeffreestar	12,816
2	@ebay	194,975	27	@sidharth_shukla	12,591
3	@youtube	141,356	28	@rubidilaik	12,017
4	@etsy	89,344	29	@potus	12,014
5	@realdonaldtrump	54,010	30	@hwanniepromotes	11,672
6	@ishehnaaz_gill	48,226	31	@ladyincrypto	10,052
7	@missufe	33,847	32	@weareoneexo	9932
8	@chitaglorya__	29,150	33	@barackobama	9855
9	@bts_twt	28,034	34	@originalfunko	9700
10	@maymayentrata07	27,304	35	@gemhostofficial	9549
11	@bloglovin	20,669	36	@colorstv	9385
12	@zazzle	18,945	37	@nytimes	8983
13	@pledis_17	18,372	38	@taylorswift13	8809
14	@joebiden	17,717	39	@cashapp	8526
15	@pulte	17,515	40	@shill_ronin	8336
16	@blackpink	16,611	41	@bang_garr	8062
17	@eyehinakhan	16,395	42	@prctiu	7762
18	@sof1azara03	16,147	43	@influenster	7589
19	@davelackie	14,343	44	@elonmusk	7452
20	@fineartamerica	14,292	45	@perduechicken	7404
21	@etsysocial	14,251	46	@netflix	7366
22	@barber_edward_	14,115	47	@colourpopco	7242
23	@cnn	13,872	48	@thesecret	7191
24	@amazon	13,285	49	@kamalaharris	7187
25	@giveawayhost	13,275	50	@taegiveaway	7171

Regarding the influential actors, we take two approaches. The first one is to simply identify what user accounts are mentioned the most in the tweets, which can be considered to be the popular users in this virtual community. Table 5 presents the top-50 popular user mention ranking from the tweets posted by the same young female users interested in fashion. The user @poshmarkapp is the most mentioned user account, which confirms that shopping on Poshmark is very popular. Note that not all the user accounts listed on this ranking match the young female users in our target audience. They are just the user accounts that were mentioned very frequently by them, some of whom can be outside the target audience.

The second approach to identifying influencers is to leverage two commonly-used measures: eigenvector centrality ([79]) from the network theory and retweet h-index from [80], which is an adaptive version of the traditional Hirsch index to retweets in Twitter data. For the eigenvector centrality measure, we first collect followers and followees data using the Twitter API mentioned in "User profiling" section, identify mutually following pairs of the young female users, and then build an undirected network graph. The network has 9809 nodes, which means that 9809 users out of 16,011 are connected to at least one user. This network is much denser than expected, considering that the users do not share many attributes: they only share the interest in fashion, the gender, and the age class. We finally apply the eigenvector centrality algorithm to the network graph, which basically favors users who are connected with other well-connected users in the network. This results in a centrality score for each user in the graph. It turns out that most users have very low centrality scores, whereas only a few have high centrality scores. We believe that this demonstrates a good example of the existence of influencers in a certain domain. For the retweet h-index measure, we use the **Tweet** object that contains the information of how many times a tweet has been retweeted by other users. This also results in a retweet h-index value for each user.

**Table 6:** Top-25 influential actors among the young female users interested in fashion sorted by centrality (left side) and h-index (right side), respectively, in descending order

Rank	User	Centrality	User	H-Index
1	@jacquelinerline	0.124	@makeupbyshaniah	191
2	@ofresell	0.105	@nikkitamboli	177
3	@captaincouture1	0.099	@c**********s	174
4	@heliapichardo	0.098	@m********x	171
5	@bethpaintings	0.098	@josinaanderson	161
6	@katewinstyle	0.097	@alissawahid	156
7	@trixie8181	0.095	@janeyellene	140
8	@pinkpretty16	0.094	@salmahayek	140
9	@lashea_hudnall	0.094	@g*************1	137
10	@amyposhboutique	0.091	@rubidilaikofc	135
11	@msmaverick2	0.09	@megastyleph	133
12	@micely6391	0.088	@maliibumiitch	123
13	@peanutandjojos	0.088	@ari_maj1	118
14	@chelleztreasure	0.088	@nikkisamonas	116
15	@emmasattic98	0.088	@rubiholiccs	114
16	@suzcat12	0.087	@emilykschrader	112
17	@jazziesposhmark	0.087	@famnikki	111
18	@poshmarkrebekah	0.086	@ivy_ferguson	108
19	@lifesshortbuyit	0.085	@s*************s	107
20	@shadowdogdesign	0.08	@sayyess2thejess	105
21	@rendon_patsy	0.077	@aquiboni	102
22	@krista47005550	0.076	@life_breakdown	102
23	@boondockfinds	0.075	@shivandi	98
24	@voudaux	0.075	@hinakhanstan	96
25	@michelleroseg33	0.073	@a************o	93

Table 6 presents the top-25 influential user ranking sorted by centrality (left side) and h-index (right side), respectively, in descending order. The number one user on the centrality ranking is Jacqueline Line (screen name @JacquelineRLine), who has 367K followers at the time of writing, is a popular user on Poshmark, and her timeline is filled with tweets on various fashion items. On the other hand, the number one user on the retweet h-index ranking is Shaniah (screen name @makeupbyshaniah), who has 115.4K followers at the time of writing, is a popular makeup artist and YouTuber. As shown in the table, the two influencer rankings present completely different users, which implies that the two measures exhibit different perspectives on influence.^14^

It is worth further analyzing this case study from a perspective of the Total Twitter Error framework mentioned in "Introduction" section, which helps us to evaluate potential errors in the study. As the study completely relies on the pool of random Twitter users and tweets to identify people interested in fashion, it is not free from the under-coverage error. In other words, it is obvious that the Twitter users found never represent all people in the world interested in fashion. Here, we make a strong assumption that we are only interested in Twitter users and our study is only targeted at those people in a social media world. We do not believe that this assumption is unreasonable, as we are well-aware that many people interested in fashion are using Twitter and having conversation in the cyberspace. Again, this should completely depend on the objectives of the study. On the other hand, the 16,011 young female users found are never small as a sample, as it would be challenging to gather this number of human subjects or respondents in traditional surveys. In addition, we identified and removed organization accounts, which definitely helped to reduce the over-coverage error in our data. In terms of the query error, while we could have added other hashtags than just #fashion and #style when identifying users interested in fashion, we believe that the two hashtags alone are representative of the interest in fashion. Lastly, there is room for the interpretation error, given that the user profiling solutions used are imperfect. In order to minimize the potential interpretation error, we (1) chose the solutions that demonstrate good performances in their papers and also (2) used more than one solutions for the same attribute whenever possible.

One limitation in this case study is that it would be ideal if we could compare the trends observed on Twitter to actual observable indicators coming from out-of-Twitter. To the best of our knowledge, we are unaware of any external data sets that can be mapped to our topic and user rankings for cross-evaluation. This limitation suggests future research in this case study.

### Me Too movement reaction: conservatives vs. liberals

The second case study aims to answer the question of whether the political orientation, i.e., conservative vs. liberal, affects people’s reaction to a gender-related issue. We choose the recent Me Too movement as one of the noticeable gender-related topics and attempt to compare how differently conservatives and liberals react to the same issue. To define the target audience for this case study, we take the same approach as the one used in the previous case study on young women interested in fashion: identifying the Twitter users in our pool who have ever used the #metoo hashtag in their tweets. Again, mentioning the hashtag is assumed to be their interest in the topic. From our pool, 68,116 users are identified as those who (1) have ever used the #metoo hashtag, (2) still have valid and public accounts on Twitter, and (3) have posted at least 100 tweets. Formally, $|U| = 68116$. We then collect up to 3200 recent tweets for each of the users, which totals 188,806,239 tweets, or formally $|T| = 188806239$.

The next step is to partition the users into two groups: conservatives and liberals. To that end, we opt to develop our own hashtag-based political orientation classifier fitted to our Twitter data for the same reason stated in "Customized user profiling" section. Specifically, we collect another set of users who can be easily labeled as “conservative” or “liberal” and use hashtags of those users as the features for political orientation prediction. We again search our random pool of users and tweets for users who described themselves in their bio as “proud republican”, “proud conservative”, “proud democrat”, or “proud liberal”, based on the observation that these expressions are a common way of expressing one’s political orientation and thus can serve as a strong indicator of their political orientation. In this way, we find the users who have those proud republican or conservative expressions in their bio and label them as “conservative”. Similarly, we label those who describe themselves as proud democratic or liberal as “liberal”. We further check if (1) the users still have valid and public accounts on Twitter and (2) have posted at least 100 tweets. This leaves 5,740 users in total, of which 4717 users are labeled as “liberal” and 1023 users are labeled as “conservative”. We then collect up to 3200 recent tweets of the users, which results in 12,299,722 tweets in total. From the collected tweets, we now extract top-1000 popular hashtags, which will be used as the features for prediction. As in the first case study, hashtags exclusively used by a single user are excluded. Table 7 presents the top-50 popular hashtags. As shown in the table, most of the hashtags are directly or indirectly related to politics, which is a clear indication that the labeled users collected for machine learning are interested in politics. Many of the hashtags on the ranking appear to be discriminative between the two classes, conservative and liberal, such as #trump and #bidenharris2020.

**Table 7:** Top-50 popular hashtags from the tweets posted by the users labeled as “conservative” or “liberal”

Rank	Hashtag	Frequency	Rank	Hashtag	Frequency
1	#covid19	12,753	26	#imwithher	2541
2	#trump	10,706	27	#strongertogether	2524
3	#resist	6515	28	#biden2020	2502
4	#maga	6223	29	#trumpvirus	2382
5	#fbrparty	5979	30	#tiktok	2292
6	#bidenharris2020	5941	31	#trump2020	2266
7	#potus	5796	32	#resisters	2262
8	#fbr	5274	33	#buildbackbetter	2248
9	#backfiretrump	4943	34	#votebluetosaveamerica	2200
10	#vote	4915	35	#florida	2165
11	#breaking	4684	36	#traitortrump	2161
12	#fbi	4564	37	#lockhimup	2157
13	#theresistance	4061	38	#trumpcrimefamily	2153
14	#moscowmitch	3801	39	#poshmark	2133
15	#coronavirus	3752	40	#biden	2083
16	#mitchplease	3429	41	#trumprussia	2067
17	#gop	3157	42	#auschwitz	1954
18	#blacklivesmatter	3073	43	#scotus	1904
19	#smartnews	2826	44	#demdebate	1895
20	#voteblue	2770	45	#giveaway	1854
21	#newprofilepic	2706	46	#resistance	1840
22	#demvoice1	2631	47	#georgia	1834
23	#covid	2591	48	#texas	1826
24	#gh	2570	49	#txlege	1815
25	#impeachtrump	2546	50	#sotu	1777

In our data, there are more samples tagged with “liberal” (4717) than those with “conservative” (1023). To avoid any potential bias in the classifier, we transform this unbalanced data set into a balanced data set by undersampling, i.e., selecting the same number of random samples from “liberal” samples as “conservative” samples. Next, we randomly split this data set of equal numbers of “conservative” and “liberal” samples into 80% of training data (1636 samples) and 20% of test data (410 samples). Then, to build a classification model, we apply widely-used classification algorithms to the training data, such as k-Nearest Neighbors, Logistic Regression, Random Forest, XGBoost, Support Vector Machines, Neural Networks, and Deep Neural Networks, for each of which we find the best hyper-parameters that yield the best performance. Lastly, we evaluate each model on the test data.

**Fig. 2:**
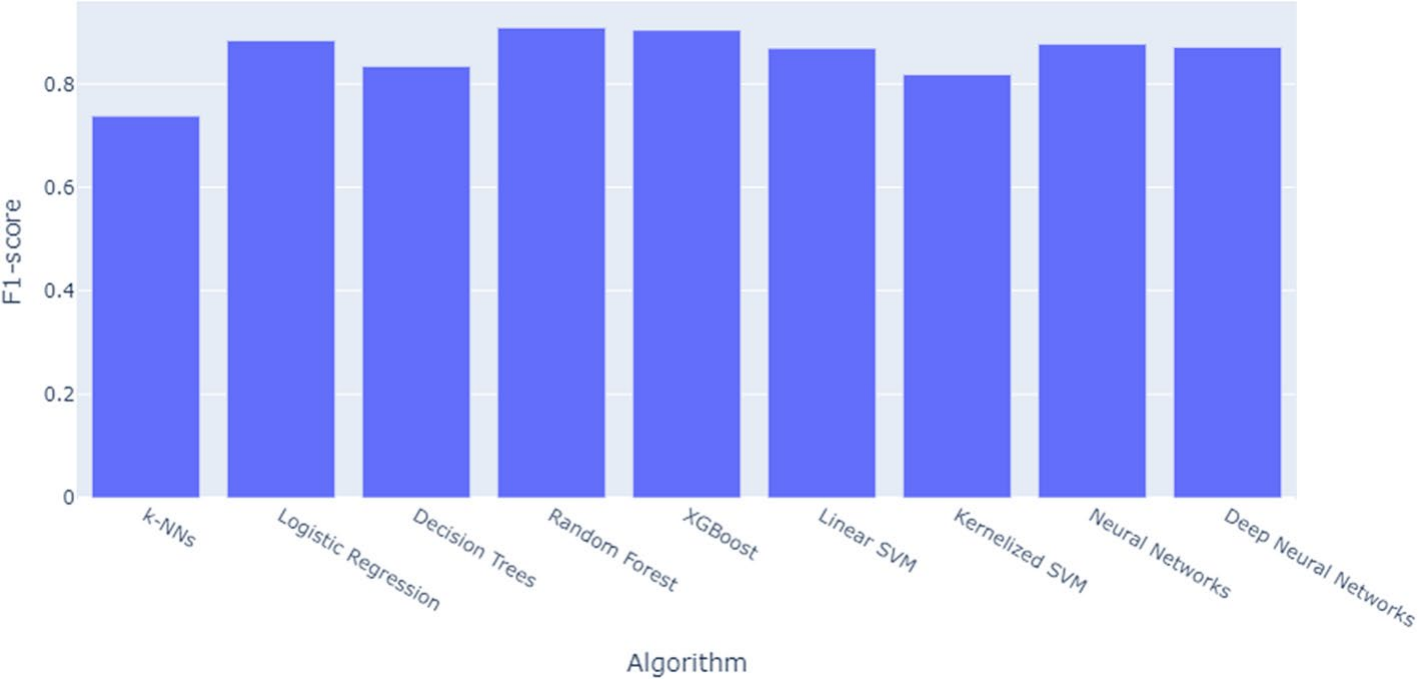
Comparison of f1-scores for the nine classification algorithms

**Fig. 3:**
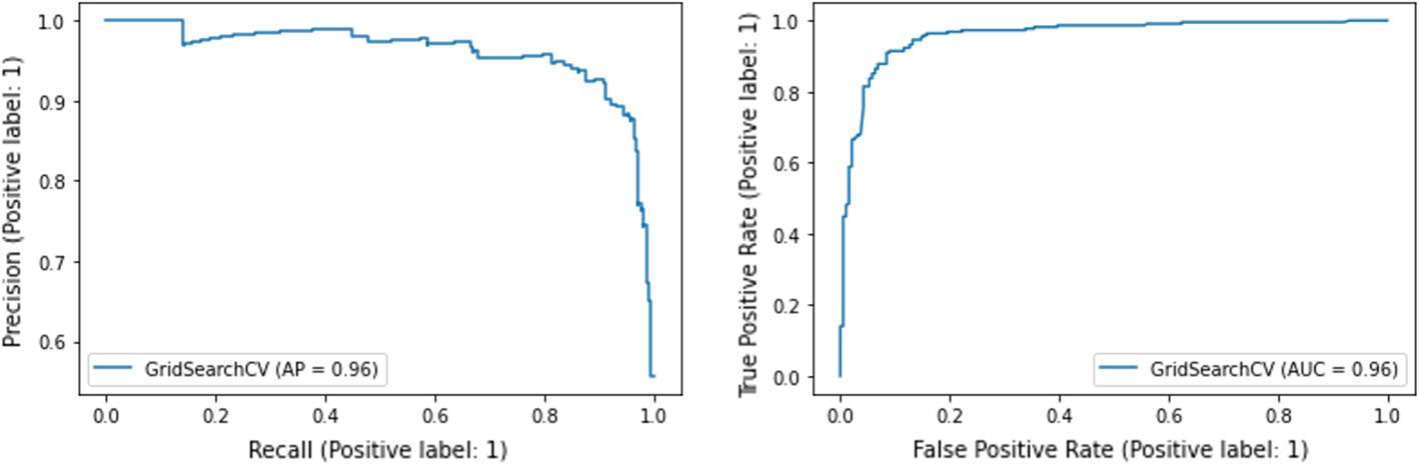
The Average Precision (AP) curve (left) and the Receiver Operating Characteristic (ROC) curve (right) for the best performing Random Forest model

**Table 8:** Top-50 important features for the best performing political orientation classifier using the Random Forest﻿ algorithm

Rank	Feature	Importance	Rank	Feature	Importance
1	#trump2020	0.042	26	#fbrparty	0.008
2	#fjb	0.038	27	#trumpshutdown	0.008
3	#moscowmitch	0.034	28	#impeachtrump	0.008
4	#traitortrump	0.029	29	#neverforgetjanuary6th	0.008
5	#oann	0.026	30	#deathsantis	0.007
6	#resist	0.021	31	#expeljoshhawley	0.007
7	#bidenharris2020	0.020	32	#daytona500	0.007
8	#americafirst	0.019	33	#fbi	0.006
9	#voteblue	0.017	34	#prolife	0.006
10	#2a	0.015	35	#wearamask	0.006
11	#bidenharris	0.015	36	#trump2024	0.006
12	#istandwithbiden	0.015	37	#covid19	0.006
13	#demvoice1	0.014	38	#proudboys	0.006
14	#mitchplease	0.014	39	#laurenboebertissodumb	0.005
15	#getvaccinated	0.012	40	#resisters	0.005
16	#buildbackbetter	0.012	41	#trumpvirus	0.005
17	#forthepeople	0.011	42	#votebluetosaveamerica	0.005
18	#theresistance	0.011	43	#morningjoe	0.005
19	#godblessamerica	0.011	44	#strongertogether	0.005
20	#walkaway	0.011	45	#lockhimup	0.005
21	#trumpisnotwell	0.010	46	#americasgreatestmistake	0.005
22	#antifa	0.010	47	#trumpcare	0.005
23	#maddow	0.010	48	#holocaustremembranceday	0.005
24	#arresttrumpnow	0.010	49	#trumprussia	0.005
25	#backtheblue	0.009	50	#maga2020	0.005

For model evaluation and selection, we compare the f1-scores, which are the harmonic means of precision and recall. As shown in Fig. 2, the Random Forest model yields the best performance with the f1-score of 0.91, which can be considered a very high accuracy for prediction. Figure 3 presents the Average Precision (AP) curve (left) and the Receiver Operating Characteristic (ROC) curve for the best performing Random Forest model. The Average Precision and Area Under the Curve (AUC) are 0.96 and 0.96, respectively, which confirm the excellent performance of the model. In addition, in order to identify which features (i.e., hashtags) contribute the most to prediction, we list the feature importance scores provided by the Random Forest algorithm. Table 8 presents the top-50 important features and their importance scores. The ranking shows that the #trump2020 hashtag contributes the most in terms of political orientation prediction, followed by #fjb, #moscowmitch, #traitortrump, #oann (meaning One America News Network), #resist, #bidenharris2020, and so on, which all make sense.

As the training data used for political orientation classification are biased toward the users who clearly described themselves as proud liberal/conservative, we further conduct out-of-sample performance evaluation. To create a new data set for out-of-sample evaluation, we randomly select 200 users whose bio has “democrat” or “liberal” with no “proud” and, likewise, 200 users whose bio has “republican” or “conservative” with no “proud”. Next, for each of the group of 200 users, we manually check if the user is actually liberal or conservative by reading their bio, which results in 179 liberal users and 116 conservative users. We then collect up to 3200 most recent tweets from their timelines and extract hashtag frequency features from their tweets. We then apply our political orientation classifier to those users and predict their political orientations. Finally, we compare their predicted political orientations with their actual ones. This results in an f1-score of 0.76. While this performance is lower than the with-in sample performance of 0.91, which is fully expected, the performance is still high enough to be used in real-world Big Data analysis.

In order to prove that hashtag features outperform full-text features in political orientation classification, we utilize BERT ([81]) as the baseline approach to compare, which is known to perform well in text classification. To clarify, our approach uses the frequencies of top-1000 popular hashtags as features, whereas BERT uses the full text of aggregated tweets of users as features for transfer learning. The f1-score we achieve from BERT is 0.61, which is far lower than 0.91 from the best-performing hashtag-based model. Our guess is that the full text of a user’s tweets has too much noise that does not help in identifying their political orientation, whereas hashtags serve as surprisingly good indicators.

Now that we have our own political orientation classifier fitted to tweet data, we apply the classifier to our 68,116 users who are interested in #metoo. This results in 46,037 users labeled as “conservative” and 22,079 users labeled as “liberal”. Unlike the training and test data for modeling the classifier, there are more conservatives than liberals in our Me Too data set.

**Table 9:** Comparison of the top-50 popular hashtags from the #metoo tweets posted by the users labeled as “liberals” and by “conservatives”, respectively

Rank	Liberals		Conservatives	
	Hashtag	Frequency	Hashtag	Frequency
1	#metooindia	2497	#timesup	2151
2	#timesup	1909	#metooindia	1579
3	#metoogr	1314	#blm	865
4	#ge	1042	#occupy	741
5	#firstthem	787	#metoogr	712
6	#metooincest	729	#believewomen	706
7	#metooinceste	666	#ibelievetarareade	679
8	#india	529	#daca	662
9	#veterans	498	#demexit	652
10	#rape	455	#union	650
11	#believewomen	432	#oligarchs	650
12	#metoounlessitsbiden	419	#megabanks	650
13	#domesticviolence	368	#corpmedia	650
14	#rapeculture	358	#nodapl	650
15	#tarareade	342	#sdf	650
16	#saraheverard	322	#humanity	649
17	#sexualassault	313	#idiocracy	638
18	#doctorsaredickheads	291	#ibelievetara	605
19	#weasourselves	286	#timesupbiden	478
20	#blacklivesmatter	281	#maketellingsafe	473
21	#mentoo	278	#csa	469
22	#silenceisviolence	275	#dropoutbiden	469
23	#doctorsabusetoo	270	#metoounlessitsbiden	445
24	#blm	265	#firstthem	437
25	#patientchoice	262	#mentoo	407
26	#nursesabusetoo	262	#dropbiden	373
27	#metoocy	259	#feminism	366
28	#anopensecret	252	#tarnishedbadge	363
29	#believeallwomen	246	#auspol	334
30	#justiceforjohnnydepp	242	#whyididntreport	318
31	#ibelievetarareade	232	#blacklivesmatter	282
32	#violenceagainstwomen	229	#women	280
33	#churchtoo	219	#bjp	274
34	#joebiden	214	#kobebryant	266
35	#h1news	193	#koberip	264
36	#women	188	#feminist	259
37	#sexualharassment	187	#believesurvivors	258
38	#feminism	185	#joebidenisarapist	244
39	#ibelievetara	182	#biden	241
40	#metoomovement	173	#feminismiscancer	240
41	#patientdignity	173	#bringbernieback	238
42	#notallmen	165	#endviolenceagainstwomen	235
43	#covid19	164	#justice	233
44	#unstucklife	164	#survivors	227
45	#china	163	#covid19	226
46	#hr	154	#neverbiden	222
47	#awareness	151	#book	205
48	#survivor	149	#survivor	204
49	#biden	144	#london	200
50	#anuragkashyap	143	#brexit	199

We now proceed with the final step for comparing the views on the Me Too movement by political orientation. We compare the most popular hashtags that co-occur with the #metoo hashtag in the same tweet, based on the idea that there would be differences between liberals’ interests and conservatives’ interests in the same Me Too context. Table 9 presents the top-50 popular hashtag rankings from the tweets posted by liberals and by conservatives, respectively. Note that, while this table only shows the 50 most popular hashtags, there are much more hashtags following those top-50 hashtags.

In order to measure how different the two entire rankings are, we employ two measures: the cosine similarity and the rank correlation. For the cosine similarity measure, specifically, we transform each entire ranking into a vector of hashtag frequencies and then calculate the cosine similarity between the two vectors, which indicates the angle between the two vectors. The smaller the angle, the more similar the two vectors are. Cosine similarity ranges between 0 and 1, where being close to 1 means very similar and being close 0 means dissimilar. From the two hashtag ranking vectors, we get the cosine similarity of 0.65. For the second rank correlation coefficient measure, we calculate both the Spearman correlation coefficient and the Kendall correlation coefficient on the two entire rankings. A rank correlation coefficient ranges from −1 and 1, where being close to 1 indicates a positive correlation, being close −1 a negative correlation, and being close to 0 no correlation. We achieve −0.24 and −0.23, respectively, which are both closer to 0 than to 1 or −1. The cosine similarity and the rank correlation coefficients indicate the dissimilarity of the two rankings, which implies that the two groups’ interests are not the same.

**Fig. 4:**
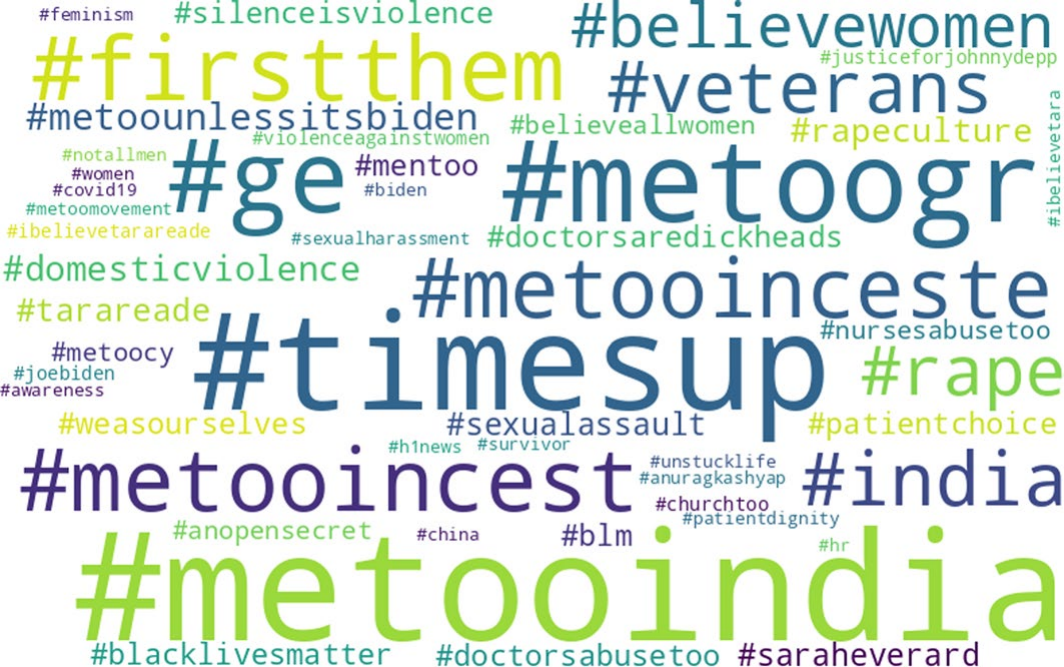
Top-50 popular hashtags used by the users labeled as “liberal” in #metoo tweets

**Fig. 5:**
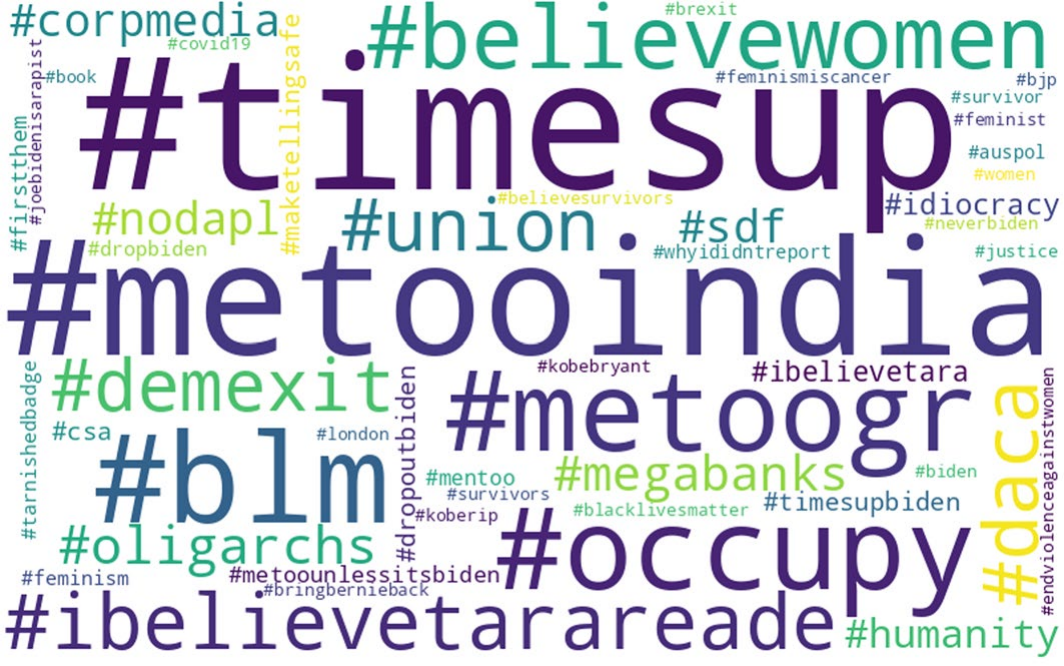
Top-50 popular hashtags used by the users labeled as “conservative” in #metoo tweets

To get an idea of specifically how the two rankings are different, Figs. 4 and 5 present the top-50 popular hashtag clouds for the liberals and the conservatives, respectively, in which larger hashtags represent more popular ones. Noticeably, the two hashtag clouds present somewhat different hashtags, as they have only 16 hashtags in common.^15^ Besides, many of the hashtags do not appear on the other cloud.^16^ These all confirm that liberals and conservatives do not equally take the same gender-related issue showing interests in somewhat different topics.

We now evaluate potential errors in this case study from a Total Twitter Error perspective. As with the first case study, this study relies on the pool of random Twitter users and tweets to identify people interested in the Me Too movement, and thus the same argument holds for this study: we assume that the set of 68,116 Twitter users found is sufficient for the study. In terms of the query error, we believe that the #metoo hashtag is the one and only hashtag we can think of and is representative of the interest in the Me Too movement, although there is a possibility that some users did not use the #metoo hashtag in their tweets. In this case, one may consider searching for any other expressions than just hashtags in tweet text that represent Me Too. Lastly, given the very high accuracy of our political orientation classifier, we believe that there is not much room for the interpretation error caused by customized profiling.

## Discussion and conclusions

In this paper, we develop a generic procedure that enables researchers to discover social trends from the collective voice of target users on Twitter. Our proposed approach provides a comprehensive guidance on how to identify a target audience of users on Twitter and discover social trends represented by hashtags, which we believe are unique and hard to acquire otherwise. We choose Twitter among many other social media platforms primarily due to its open and data-friendly nature which has attracted a large number of not only people as its users but also researchers who are interested in public opinions and social trends. We first address the problem of identifying the right users that meet certain criteria from a large pool of random Twitter users, leveraging a wide range of user profiling techniques proposed to date for many different purposes. If the basic user profiling is not satisfactory, we propose to, when possible, consider customized user profiling by developing a machine learning solution to a specific user profiling task. Once the target users have been identified, we explore mining hashtags from the tweets created by the users. Our findings from the two in-depth case studies, one on women interested in fashion and the other on people who reacted to the Me Too movement, demonstrate that the findings acquired by our approach offer unique perspectives and opportunities for social trend analysis.

There is a potential limitation of this work, which we call the target user update problem. While there are user attributes that are less subject to change such as gender, race/ethnicity, and personality traits, some of the attributes are prone to change such as location and interest. Furthermore, Twitter users can update their profiles, which can lead to a case in which some users are identified as having a certain attribute value based on their bio at some point, but at a later point they are no longer identified as having the attribute value, because they have changed their bio. This could be critical to a study, considering the fact that some studies aim to track a social trend over time, and therefore those users who are inaccurately identified as target users may continue to have a negative impact on the analysis. This is a good example of the coverage error mentioned by Hsieh et al. [6]. In this case, a decision needs to be made on whether to embrace them throughout the study or update the users at every time point. When updating the users, one should be aware that it requires an update of the entire tweet data including hashtags, which can result in a new version of customized user profiling, which can also lead to different user attribute values.

It is worth mentioning that our proposed method for customized user profiling does not work for all cases. It specifically relies on the hashtags used by the users and is limited to a classification task for user profiling. Nevertheless, we believe that it is useful for many cases, considering the fact that many of the user profiling tasks deal with classification as with gender or political orientation classification, and that it can be a good complement to the available solutions that fail to fill a user attribute of all users. We also acknowledge that the current study is only a starting point that can lead to more interesting and deeper research on text analysis in a variety of disciplines.

## Data Availability

The data and the code are available upon request.

## Abbreviations

API: Application Programming Interface
MBTI: Myers-Briggs Type Indicators
JSON: JavaScript Object Notation
NLP: Natural Language Processing
AP: Average Precision
ROC: Receiver Operating Characteristic
AUC: Average Precision and Area Under the Curve
BERT: Bidirectional Encoder Representations from Transformers

## Footnotes

1 Twitter API [website], https://developer.twitter.com/en/docs/twitter-api, (accessed February 1, 2022).

2 Filter realtime tweets [website], https://developer.twitter.com/en/docs/twitter-api/v1/tweets/filter-realtime/overview, (accessed February 1, 2022).

3 Twitter objects and fields are discussed in detail in "User profiling" section.

4 Introducing JSON [website], https://www.json.org/, (accessed February 1, 2022).

5 Data dictionary: Standard v1.1 [website], https://developer.twitter.com/en/docs/twitter-api/v1/data-dictionary/overview/user-object, (accessed February 1, 2022).

6 Follow, search, and get users [website], https://developer.twitter.com/en/docs/twitter-api/v1/accounts-and-users/follow-search-get-users/api-reference/get-followers-ids; https://developer.twitter.com/en/docs/twitter-api/v1/accounts-and-users/follow-search-get-users/api-reference/get-friends-ids, (accessed February 1, 2022).

7 About Verified Accounts [website] https://help.twitter.com/en/managing-your-account/about-twitter-verified-accounts, (accessed February 1, 2022).

8 Data dictionary: Standard v1.1 [website], https://developer.twitter.com/en/docs/twitter-api/v1/data-dictionary/overview/tweet-object, (accessed February 1, 2022).

9 Get Tweet timelines [website], https://developer.twitter.com/en/docs/twitter-api/v1/tweets/timelines/api-reference/get-statuses-user_timeline, (accessed February 1, 2022).

10 Follow, search, and get users [website], https://developer.twitter.com/en/docs/twitter-api/v1/accounts-and-users/follow-search-get-users/api-reference/get-users-lookup, (accessed February 1, 2022).

11 Humanizr: Bringing the humanity back to Twitter [website], https://github.com/networkdynamics/humanizr, (accessed February 1, 2022).

12 M3-Inference [website], https://github.com/euagendas/m3inference, (accessed February 1, 2022).

13 gender-guesser 0.4.0 [website], https://pypi.org/project/gender-guesser/, (accessed February 1, 2022).

14 One issue we have with the retweet h-index measure is that some of the top influencers from h-index post sexually-explicit content on their timelines. As such, we mask the screen names of those five users in the table in order to avoid readers’ unexpected exposure to that inappropriate content.

15 The two hashtag clouds have the following hashtags in common: #believewomen, #biden, #blacklivesmatter, #blm, #covid19, #feminism, #firstthem, #ibelievetara, #ibelievetarareade, #mentoo, #metoogr, #metooindia, #metoounlessitsbiden, #survivor, #timesup, and #women.

16 The hashtags that appear on the liberals ranking but not on the conservatives include #anopensecret, #anuragkashyap, #awareness, #believeallwomen, #china, #churchtoo, #doctorsabusetoo, #doctorsaredickheads, #domesticviolence, #ge, #h1news, #hr, #india, #joebiden, #justiceforjohnnydepp, #metoocy, #metooincest, #metooinceste, #metoomovement, #notallmen, #nursesabusetoo, #patientchoice, #patientdignity, #rape, #rapeculture, #saraheverard, #sexualassault, #sexualharassment, #silenceisviolence, #tarareade, #unstucklife, #veterans, #violenceagainstwomen, and #weasourselves. Those that appear on the conservatives ranking but not on the liberals ranking include #auspol, #believesurvivors, #bjp, #book, #brexit, #bringbernieback, #corpmedia, #csa, #daca, #demexit, #dropbiden, #dropoutbiden, #endviolenceagainstwomen, #feminismiscancer, #feminist, #humanity, #idiocracy, #joebidenisarapist, #justice, #kobebryant, #koberip, #london, #maketellingsafe, #megabanks, #neverbiden, #nodapl, #occupy, #oligarchs, #sdf, #survivors, #tarnishedbadge, #timesupbiden, #union, and #whyididntreport.
